# Physical Activity, Blood Pressure, and Community Dining Sites: a Cohort Study of Older Black Adults Who Participate in the Congregate Meal Program

**DOI:** 10.1007/s40615-025-02664-7

**Published:** 2025-09-29

**Authors:** Martha Y. Kubik, Niloofar Ramezani

**Affiliations:** 1 College of Public Health, George Mason University, 4400 University Drive, MSN 3C4, Fairfax, VA 22030 USA; 2 School of Public Health, Virginia Commonwealth University, Richmond, VA 23284 USA

**Keywords:** Physical activity, Blood pressure, Older Black adults, Social determinants of health

## Abstract

**Objective:**

Hypertension especially systolic hypertension is common in older adulthood and disproportionately affects Black adults. Physical activity (PA) contributes to improved blood pressure (BP) and cardiovascular health. However, opportunities for PA for older adults are often limited.

**Methods:**

We used a prospective observational design and data collected fall 2022 (time-1) and spring 2023 (time-2) from Black adults (n = 177) ≥ 60 years-of-age participating in the congregate meal program at community dining sites in Washington DC. Sites included senior centers, park/recreation facilities, and public housing. Measured height/weight/BP and a self-report survey were collected, with PA measured using the 9-item Rapid Assessment of Physical Activity, validated for use in older adults. Chi-square tests compared participant’s PA levels by dining site. Hierarchical, repeated-measures linear regression was used to examine the association between PA at time-1 and systolic blood pressure (SBP) at time-2.

**Results:**

Most participants were female (78%), lived alone (72%), with 52% reporting ≤ high school education. Mean (SD) age was 75 (8) years. Mean (SD) SBP was 138 (20) mm Hg. Mean (SD) diastolic BP was 70 (11) mm Hg. Most (75%) self-reported hypertension. PA varied by dining site, with lowest rates reported by participants at public housing sites (*p* < 0.001). PA was inversely related to SBP (Beta -1.01; 95% CI -1.94, -0.09; *p* = 0.03).

**Conclusions:**

Study findings support the benefit of PA to lower SBP among an older Black adult population and suggest increasing PA programming at community dining sites may be one strategy to improve PA and cardiovascular health. Further study is warranted.

## Introduction

With an aging adult population, renewed attention to the prevention and management of hypertension, a major modifiable risk factor for cardiovascular disease, is merited. Hypertension is common in older adulthood, particularly systolic hypertension, affecting more than 75% of adults age 65 and older [1, 2]. In the US, the burden of hypertension is disproportionately experienced by Black adults who are more likely than White adults to have poorly managed hypertension and experience higher rates of hypertension-related morbidity and mortality, including fatal stroke, cardiovascular mortality and end-stage renal disease [3, 4]. A robust literature supports the benefits of physical activity (PA) to lower blood pressure (BP) and reduce cardiovascular risk [1]. However, only 23% of adults age 65 and older meet federal PA guidelines (150 min of moderate or 75 min of vigorous aerobic activity and two days of muscle strengthening per week) [5], with almost one-third physically inactive (no physical activity or exercise other than their regular job in the past 30 days) [6].

Among factors that contribute to disparities in hypertension prevalence and management among Black as well as other minoritized groups is the unequal distribution of social determinants of health that include neighborhood conditions that limit opportunities to be physically active and connection with others for social support [4, 7]. For an aging population, one strategy to address barriers to physical activity is to identify and maximize use of community resources already in place and accessed by older adults [7]. The congregate meal program, with funding from the Older Americans Act, provides healthy meals and an opportunity for socialization to an annual average of 1.6 million older adults in a variety of community-located dining sites across the US [8]. Expanding services at dining sites to routinely include PA programming would afford large numbers of older adults, many with financial and social need, an opportunity to be active in an accessible, familiar and supportive environment. For older Black adults, access to PA programming at community dining sites may be particularly beneficial and contribute to increasing PA levels and improving BP control. However, research describing congregate meal program participants has not typically included an assessment of PA levels or measured BP [9]. Furthermore, research identifying promising community practices that meet the needs of specific populations who face disparities, such as older Black adults, is limited [4, 7].

Therefore, the aims of the current study were twofold and included 1) assessing BP and PA levels of older Black adults who visit community dining sites and participate in the congregate meal program and 2) evaluating the association between PA and change in systolic blood pressure (SBP) over time. We hypothesized that increasing levels of PA would be associated with decreasing SBP, with PA levels varying by the PA opportunities available at community dining sites.

## Methods

### Design, Setting, and Sample

The current secondary analysis used a prospective observational design and data collected from the ageWELL DC proof of concept study, conducted in Washington DC (2022–2023) [10]. The proof of concept study included a health and wellness program delivered weekly by faculty-supervised, prelicensure nursing students for 10–12 weeks. A convenience sample of adults age 60 years and older who participated in the federally funded congregate meal program offered at six community dining sites, including two senior centers, two park/recreation facilities and two public housing sites volunteered for study participation. Two sites were randomly selected to receive the health and wellness program while the other sites continued usual programming. As part of the proof of concept study, we analyzed select health outcomes, which included SBP and PA, and found no statistically significant treatment effects.

Data collection occurred in fall 2022 (time-1) and spring 2023 (time-2), with participants receiving a $20 and $30 gift card, respectively, for completing measurement. Trained research staff measured BP and height/weight and administered an electronic self-report survey, using standardized procedures [11, 12]. Participants provided written informed consent prior to time-1 data collection. The lead author’s Institutional Review Board approved the study protocol. A total of 189 participants provided data at both timepoints. For the current study, only participants who self-identified as Black race (*N* = 177) were included in the analyses.

### Measures

BP measurements were obtained with a mercury sphygmomanometer, using two readings to determine mean BP. Height and weight were collected in duplicate and averaged. BMI was then calculated as weight(kg)/[height(m)] ^2^. Select items from the self-report survey were also assessed.

PA was measured using the Rapid Assessment of Physical Activity (RAPA), a 9-item questionnaire validated for use in older adults [13]. The RAPA includes two parts, with yes/no response options for all items. The 7-item RAPA-1 assesses quantity and intensity of aerobic activity, scored on a 1/sedentary to 7/vigorous scale, with scores of 6 or 7 categorized as active [13]. The 2-item RAPA-2 assesses participation in strengthening and flexibility activities [13]. For the current study, strength and flexibility were evaluated as single items, each scored as 0/no or 1/yes. For the regression analyses, a continuous PA variable was created by summing the RAPA-1 score and strength and flexibility scores, with total range 1 to 9, with higher scores better.

### Analyses

Descriptive statistics were used to describe participant characteristics. Bivariate analyses and Chi-square tests were used to examine differences in participant’s PA and other select characteristics, by dining site. Hierarchical, repeated-measures linear regression was used to examine the association between PA (time-1) and systolic BP (time-2), both modeled as continuous variables, adjusting for sex, treatment x time interaction and dining site as a random effect. Although the interaction term was not significant (*p* = 0.07), it was retained in the final model to account for any residual confounding associated with the treatment. Statistical tests were two-sided. Significance was considered at *P* < 0.05. Statistical analyses were performed using RStudio [14].

## Results

All participants self-reported Black race (*N* = 177) and most non-Hispanic ethnicity (98%). Most were female (80%) and lived alone (72%). Mean (SD) age was 75 (8) years. More than half reported a high school education (27%) or less (25%). All reported having health insurance. Mean (SD) systolic BP was 138 (20). Mean (SD) diastolic BP was 70 (11). Mean (SD) BMI was 31 (7). Hypertension (75%) was the most prevalent self-reported chronic condition, followed by high cholesterol (56%) and diabetes (29%). Most participants (78%) reported two or more chronic conditions. See Table 1.

**Table 1:** Characteristics of study participants, Fall 2022, Washington, DC (*N* = 177) ^a^

	M (SD)
Age, years	75(8)
Systolic Blood Pressure, mmHg	138 (20)
Diastolic Blood Pressure, mmHg	70 (11)
Body Mass Index ^b^	31 (7)
	No. (%)
Sex	
Female	142 (80%)
Male	35 (20%)
Race	
Black	177 (100%)
Ethnicity (n = 176)	
Non-Hispanic	172 (98%)
Hispanic	4 (2%)
Education	
< High school	45 (25%)
High school	48 (27%)
Any college/trade	84 (48%)
Lives alone	
Yes	128 (72%)
Health Insurance	
Yes	177 (100%)
Chronic conditions	
Hypertension	132 (75%)
High Cholesterol	99 (56%)
Diabetes	52 (29%)
Asthma/COPD	35 (20%)
Heart Disease	29 (16%)
Depression	25 (14%)
Stroke	17 (10%)
Kidney Disease	14 (8%)
No. chronic conditions	
0–1	39 (22%)
2–3	77 (44%)
≥ 4	61 (35%)

M(SD) Mean (Standard Deviation), No Number, COPD Chronic Obstructive Pulmonary Disease

^a^n = 177, unless otherwise specified

^b^Measured height and weight was used to calculate body mass index as weight(kg)/[height(m)] ^2^

At baseline, a small majority met guidelines for aerobic (53%), strengthening (49%) and flexibility (65%) activities. However, rates varied by dining site, with participants from public housing sites reporting rates significantly lower than other sites (*P* < 0.001). See Table 2. Prevalence of chronic conditions, number of chronic conditions, as well as BMI and systolic and diastolic BP did not vary significantly by site (data not shown). In the regression analysis, for every 1-unit increase in PA, systolic BP decreased by 1.01 mm Hg (95% CI −1.94, −0.09; *p* = 0.03). See Table 3.

**Table 2:** Prevalence of physical activity among study participants, by community dining site type, Washington, DC, Fall 2022, (*N* = 177)

Physical Activity^a^	Total (*N* = 177)	Public Housing (*n* = 74)	Senior Center (*n* = 61)	Park and Recreation (*n* = 42)	*P*^b^
	*n* (%)				
Aerobic activity					
Active	93 (52%)	22 (30%)	42 (69%)	29 (69%)	<.001
Underactive/sedentary	84 (48%)	52 (70%)	19 (31%)	13 (31%)	
Strengthening activities					
Yes	85 (48%)	22 (30%)	42 (69%)	21 (50%)	<.001
Flexibility activities					
Yes	113 (64%)	31 (42%)	53 (87%)	29 (69%)	<.001

^a^Measured with the Rapid Assessment of Physical Activity (RAPA), with aerobic activity scored on a 1/sedentary to 7/vigorous scale and scores of 6 or 7 categorized as active. Strength and flexibility were evaluated as single items, each scored as 0/no or 1/yes. The range of the summed score is 1 to 9

^b^χ^2^ tests were used for each variable to examine differences across categories. *P* <.05 was considered significant

**Table 3:** Association between physical activity and change in systolic blood pressure, by mm Hg, older Black adults (*N* = 177) *

Variable	Estimate (95% CI)	*P*-value
Physical activity (Continuous: 1–9 scale) **	−1.01 (−1.94, −0.09)	0.03
Sex (Reference: Male)	0.92 (−5.46, 7.30)	0.78
Time x Treatment Interaction ***	−5.42 (−11,21, 0.37)	0.07

mm Hg = millimeter of mercury, *CI* confidence interval

^*^ Hierarchical, repeated-measures linear regression model

^**^Aerobic activity scored on a 1/sedentary to 7/vigorous scale and scores of 6 or 7 categorized as active. Strength and flexibility were evaluated as single items, each scored as 0/no or 1/yes Range of the summed score is 1 to 9, with higher scores better

^***^ Retained in the final model to account for any residual confounding associated with the primary data and treatment effects

## Discussion

Results from the current study support a statistically significant inverse association between PA and SBP among our older Black adult sample. Further, the variability of participant PA levels seen across dining site type suggest community access to dedicated space, equipment and instructors that provide a variety of physical activities designed for an older adult population, which were routinely available at senior centers and to a lesser degree park and recreation facilities but limited at public housing sites, could result in a more active lifestyle for many older Black adults and contribute to better BP control and improved cardiovascular health.

It is not surprising that hypertension is the most common chronic disease, by far, self-reported by our study sample. However, measured BP revealed a sample mean SBP of 138 mm Hg and DBP of 70 mm Hg, which is consistent with systolic hypertension that is not controlled, defined as a SBP ≥ 130 mm Hg and DBP < 80 mm Hg [15]. While national data suggest hypertension control rates have worsened for many, disparities remain, with rates for Black adults higher when compared to other racial/ethnic groups [16]. Our data suggest this disparity likely persist into older adulthood for many Black adults.

Research shows that older adults experience cardiovascular benefits from PA that are comparable to middle-age adults, with even modest levels of PA associated with benefit when compared to being inactive [17]. However, inactivity increases substantially with advancing age, with rates highest among older adults who are Black, American Indian/Alaskan Native or Hispanic, women and those with a high school education or less [5]. While preventing inactivity among older adults is a priority, providing opportunities for those who are inactive to engage in PA is equally important. PA programs designed specifically for older adults and offered in safe, supportive, easy-to-access community settings are needed.

Historically, the Title III-C Nutrition Services Program of the Older Americans Act that funds the congregate meal program has prioritized community-located settings and services that address hunger, food insecurity, and social isolation [8]. Local service providers are encouraged to offer other health-promoting programs, with PA programming an option but not a requirement. Our study findings suggest PA programs offered at community dining sites that provide the congregate meal program are likely used by many older Black adults, especially when there are dedicated resources, such as instructors, space and equipment.

### Limitations and Strengths

Study limitations include use of a convenience sample of participants, community dining sites located in one urban area and self-reported data. There are also study strengths, which include the prospective cohort design, measured BP and a PA measurement tool validated for use in older adults. Although results are specific to Washington DC, the data add to the limited research on PA and hypertension among an older Black adult population, with consideration of social determinants of health and neighborhood assets operationalized as community dining sites serving the congregate meal program.

## Conclusions

Ongoing efforts to improve BP control among older Black adults is needed. Study findings support the benefit of PA to lower SBP among an older Black adult population and suggest increasing PA programming at community dining sites that provide the congregate meal program may be one strategy to improve PA and cardiovascular health. Further study of the proposed approach is warranted.

## Data Availability

Not applicable.
